# Body mass index is associated with region-dependent metabolic reprogramming of adipose tissue

**DOI:** 10.1016/j.bbacli.2017.05.001

**Published:** 2017-05-11

**Authors:** Marco G. Alves, Ângela Moreira, Marta Guimarães, Mário Nora, Mario Sousa, Pedro F. Oliveira, Mariana P. Monteiro

**Affiliations:** a Multidisciplinary Unit for Biomedical Research, UMIB-FCT, Institute of Biomedical Sciences Abel Salazar (ICBAS), University of Porto, Portugal; b Department of Anatomy, Institute of Biomedical Sciences Abel Salazar (ICBAS), University of Porto, Portugal; c Department of General Surgery of Hospital São Sebastião, Centro Hospitalar de Entre o Douro e Vouga, Portugal; d Department of Microscopy, Laboratory of Cell Biology, Institute of Biomedical Sciences Abel Salazar (ICBAS), University of Porto, Portugal; e Centre for Reproductive Genetics Prof. Alberto Barros (CGR), Porto, Portugal; f i3S - Instituto de Investigação e Inovação em Saúde, University of Porto, Porto, Portugal; g Obesity & Bariatric Services, University College London Hospital, London, UK

**Keywords:** ^1^H NMR, proton nuclear magnetic resonance, AT, adipose tissue, BMI, body mass index, FBS, fetal bovine serum, SAT, subcutaneous adipose tissue, VAT, visceral adipose tissue, Body mass index, Visceral adipose tissue, Subcutaneous adipose tissue, Metabolism, De novo lipogenesis, Insulin

## Abstract

Adipose tissue (AT) is involved in dysmetabolism pathogenesis. Regional fat distribution and functioning may contribute to obesity-related metabolic disorders and adverse health outcomes. Specific fat depots are suggested to possess unique biological properties, but specific metabolic profiles of subcutaneous (SAT) and visceral adipose tissue (VAT) remain unknown. We aimed to characterize VAT and SAT glucose metabolism, and their correlation with body mass index (BMI). AT samples from patients (n = 12; F:M, 9:3) with a mean age of 46 years (26–83 years) and an average BMI of 29.6 kg/m^2^ (18–37 kg/m^2^) were used. VAT and SAT explants were obtained during elective laparoscopy, either cholecystectomy for uncomplicated cholelithiasis or gastric bypass for severe obesity. Explants were placed in insulin-free cell culture media and their metabolic profile was established by proton nuclear magnetic resonance. AT explants display a glucose and pyruvate consumption and acetate production that is region-dependent according to the patients BMI. In VAT, glucose consumption was positively correlated with BMI, while alanine and lactate production were negatively correlated with BMI, whereas in SAT the patients BMI did not affect AT secretome suggesting that increased BMI promotes a metabolic reprogramming of VAT towards de novo lipogenesis. This region-dependent metabolic reprogramming of AT associated with BMI was autonomous of insulin. This data, although preliminary, suggests that there is a BMI-related remodeling of glucose metabolism in VAT. Targeting this BMI-induced metabolic shift may represent a potential target to counteract unwanted consequences derived from visceral adiposity.

## Highlights

• Metabolic profile of adipose tissue (AT) explants was studied after culture in insulin-free media.
• Visceral adipose tissue (VAT) glucose consumption was positively correlated with patient's BMI.
• Alanine and lactate production by VAT were negatively correlated with patient's BMI.
• Patient's BMI did not affect subcutaneous adipose tissue (SAT) secretome.
• BMI-related metabolic remodeling in VAT occurs beyond insulin action.

## 1 Introduction

Metabolic homeostasis depends on adipose tissue (AT) functioning. AT stores energy that can be mobilized as needed and thus, it is considered a dynamic organ with a crucial role in the pathophysiology of dysmetabolism. AT has a strong influence in glucose and lipid metabolism as well as in systemic insulin sensitivity [1]. Regional fat distribution and functioning are likely to contribute to the link between obesity, metabolic disorders and health outcomes [2], [3], [4]. Metabolic activity of visceral adipose tissue (VAT) is associated with dyslipidemia, whereas subcutaneous adipose tissue (SAT) is reported to play a pivotal role for glucose and insulin homeostasis [5]. SAT and VAT differ in the basal antioxidant status and the antioxidant response capacity during obesity [6]. In addition, excess accumulation of VAT is associated with increased risk of several health complications, including diabetes, cardiovascular disease and mortality. These deleterious effects of VAT were classically attributed to increased rates of lipolysis and release of free fatty acids into the blood stream and liver portal circulation, which would then have a negative impact on hepatic insulin sensitivity [7]. On the other hand, SAT appears to be relatively benign or even beneficial to health in some cases [8]. Using rodent models, it was shown that the transplantation of SAT to visceral locations can improve glucose metabolism and insulin sensitivity [9], illustrating that both tissues have very distinct properties from a metabolic point of view.

Excess of adipose tissue contributes to the onset and deleterious effects promoted by obesity, but distinct adipose compartments present distinct anatomic distribution, cellularity patterns and molecular functioning. In adults, > 80% of total body fat is distributed in the subcutaneous compartment while only 10 to 20% is thought to be VAT [10]. Human SAT and VAT are reported to distinctly contribute to metabolic complications. VAT has an important role in the establishment of obesity-associated health problems due to its privileged localization to portal circulation and its secretory function of key bioactive substances [11], [12]. Reducing VAT by omentectomy combined with gastric banding was also reported to have positive long-term effects on insulin sensitivity and glucose metabolic profiles of obese patients [13]. In humans, VAT is usually described as less stable and with a higher metabolic rate than SAT [14]. In addition, VAT was reported to possess higher content in metabolites involved in primary metabolism, including amino acids, carbohydrates or organic acids, while SAT was more rich in free fatty acids [15].

Though it has emerged that specific fat depots have unique biological and metabolic properties [16], [17], [18], the detailed metabolic differences between SAT and VAT remain largely unknown. To characterize the metabolic functioning of VAT and SAT and how it correlates with body mass index (BMI) is crucial to understand the pathogenesis of metabolic disorders in humans. Herein, we propose to determine VAT or SAT depots metabolism, and establish a correlation with BMI. With that purpose, paired samples of VAT and SAT tissue explants from patients undergoing elective cholecystectomy or laparoscopic gastric bypass for the primary treatment of uncomplicated cholelithiasis or obesity, respectively were cultured in the absence of insulin and their metabolic profile was established.

## 2 Material and methods

### 2.1 Chemicals

Fetal Bovine Serum (FBS) was purchased from Gibco (Life Technologies, Grand Island, NY, USA). Phenol-red free DMEM-F12, Penicillin-streptomycin (Pen-Strep) and all other chemicals were purchased from Sigma-Aldrich (St. Louis, MO, USA) unless otherwise specified.

### 2.2 Subjects

Abdominal subcutaneous and omental visceral human adipose tissue samples were collected during elective surgeries performed at Centro Hospitalar de Entre o Douro e Vouga, E.P.E, from patients without active infectious or neoplastic diseases, undergoing elective cholecystectomy or laparoscopic gastric bypass for the treatment of uncomplicated cholelithiasis or obesity, respectively. Collection of samples was performed after approval by the Local Ethics Committee and in accordance with the Guidelines of the Local, National and European Ethical Committees. All studies followed the Declaration of Helsinki for Medical Research involving Human Subjects. Patients (n = 12), were predominantly females (F:M, 9:3), with an average age of 46.8 years (range 26–83 years, mean) and an average body mass index (BMI) of 29.6 kg/m^2^ (ranging from 18 to 37 kg/m^2^).

### 2.3 Isolation and incubation of adipose tissue explants

Adipose tissue fragments from visceral and subcutaneous fat depots were collected under sterile conditions, placed in containers and subjected to identical handling. In brief, any blood and macroscopically visible damaged tissue were mechanically removed before tissue weighing. Adipose tissue fragments with similar weights were then acclimatized in fresh DMEM/F12 medium, at 37 °C, with 5% of CO_2_, for 1 h. Then, culture media was replaced by fresh DMEM/F12 media with 1% Penicillin-streptomycin without FBS. After 72 h, the explant culture media was collected for secretome metabolic analysis and the tissue was weighed.

### 2.4 Proton nuclear magnetic resonance (^1^H NMR)

The metabolite content of culture media at the end of each incubation period was determined by ^1^H NMR, as previously described [19]. Sodium fumarate at a final concentration of 1 mM was used as an internal reference (6.50 ppm) to quantify the following metabolites (multiplet, ppm): H1-α glucose (doublet, 5.22), pyruvate (singlet, 2.38), acetate (singlet, 1.9), alanine (doublet, 1.44) and lactate (doublet, 1.33 ppm). The relative areas of ^1^H NMR resonances were quantified using the curve-fitting routine supplied with the NUTSpro NMR spectral analysis program (Acorn NMR, Livermore, CA, USA). Results are expressed as nanomoles of metabolite consumed (or produced) per milligram of wet tissue.

### 2.5 Statistical analysis

All data are shown as mean ± standard error of mean (SEM). The association between metabolite consumption/production and BMI was evaluated by computing Pearson correlation coefficients (r) assuming Gaussian distribution and a confidence interval of 95%. All *P* values < 0.05 were considered statistically significant. Statistical analysis was performed using GraphPad Prism 6 (GraphPad Software Inc., San Diego, CA, USA).

## 3 Results

### 3.1 Patients serum biochemical profile

Abdominal subcutaneous and omental visceral human adipose tissue samples were collected from patients undergoing elective cholecystectomy or laparoscopic gastric bypass for the treatment of gallbladder lithiasis or obesity, respectively. The group of individuals selected presented a homogeneous serum biochemical profile, as seen in Table 1.

**Table 1:** Serum biochemical profile of the subjects included in this study.

	Mean ± SEM	Min	Max
Glycaemia (mg/dL)	100 ± 6	78	147
Insulin (μU/mL)	10.0 ± 1.3	6.4	14.7
Hb1AC (%)	5.6 ± 0.3	4.9	6.8
HOMA-IR	2.6 ± 0.5	1.6	4.9
HOMA-β	100 ± 16	58	171
Total cholesterol (mg/dL)	199 ± 18	115	247
HDL (mg/dL)	44 ± 4	31	59
LDL (mg/dL)	133 ± 13	70	158
VLDL (mg/dL)	27 ± 3	15	41
Triglycerides (mg/dL)	123 ± 12	77	158

Legend: Min – minimum; Max – maximum. SEM – Standard Error of the Mean (n = 12).

### 3.2 Human subcutaneous and visceral adipose tissue explants presented a similar metabolic profile in culture

Our data showed that glucose consumption was similar in both, VAT and SAT, which present a consumption of 246 ± 77 nmol/mg of wet tissue and 254 ± 63 nmol/mg of wet tissue, respectively (Table 2). VAT consumed 12 ± 3 nmol/mg of wet tissue of pyruvate, which was very similar to SAT that consumed 15 ± 3 nmol/mg of wet tissue (Table 2). Pyruvate is the end-product of glycolysis and can be used to either fuel mitochondria or to be converted to alanine or lactate. There was no significant increase on lactate production in SAT (86 ± 13 nmol/mg of wet tissue) when compared with VAT (59 ± 11 nmol/mg of wet tissue). The alanine production was also very similar in both tissues (Table 2). Short chain fatty acids, such as acetate, have important physiological roles in adipogenesis [20]. Our results showed that acetate production was 19 ± 7 nmol/mg of wet tissue in VAT and 24 ± 7 nmol/mg of wet tissue in SAT (Table 2).

**Table 2:** Metabolite consumption (C) and production (P) by human adipose tissue explants of visceral and subcutaneous fat.

Metabolites (nmol/mg of wet tissue)	Visceral	Subcutaneous
Glucose (C)	246 ± 77	254 ± 63
Pyruvate (C)	12 ± 3	15 ± 3
Alanine (P)	5 ± 1	8 ± 2
Acetate (P)	19 ± 7	24 ± 7
Lactate (P)	59 ± 11	86 ± 13

Legend: (C) – consumption; (P) – production. Results are expressed as mean ± Standard Error of the Mean (n = 12 for each condition).

### 3.3 Region-dependent association of glucose and pyruvate consumption by adipose tissue explants with acetate production

Our results showed that acetate production by cultured explants of VAT was positively correlated with glucose (*R* = 0.639; *p* < 0.05) and pyruvate consumption (*R* = 0.948; *p* < 0.001). No correlations were detected between glucose or pyruvate consumption by cultured explants of VAT with alanine or lactate production (Table 2). In addition, cultured explants of SAT only presented a positive correlation between pyruvate consumption and acetate production (*R* = 0.804; *p* < 0.005). No other correlation was detected between the other metabolites studied in the secretome of cultured SAT (Table 3).

**Table 3:** Correlation between glucose and pyruvate consumption (C) with the production (P) of alanine, acetate and lactate by human adipose tissue explants of visceral and subcutaneous fat.

	Visceral adipose tissue						Subcutaneous adipose tissue					
	Alanine (P)		Acetate (P)		Lactate (P)		Alanine (P)		Acetate (P)		Lactate (P)	
	R	P	R	P	R	P	R	P	R	P	R	P
Glucose (C)	↓	NS	0.639	*	↓	NS	↓	NS	↓	NS	↓	NS
Pyruvate (C)	↓	NS	0.948	***	↓	NS	↓	NS	0.804	**	↓	NS

Legend: R values are Pearson's correlation coefficient for: * *P* < 0.05; ** *P* < 0.005; *** *P* < 0.001. (C) – metabolite consumption; (P) – metabolite production; NS – non-significant.

### 3.4 Increased glucose consumption by VAT, but not by SAT, was positively associated with the BMI of the patients

**Fig. 1:**
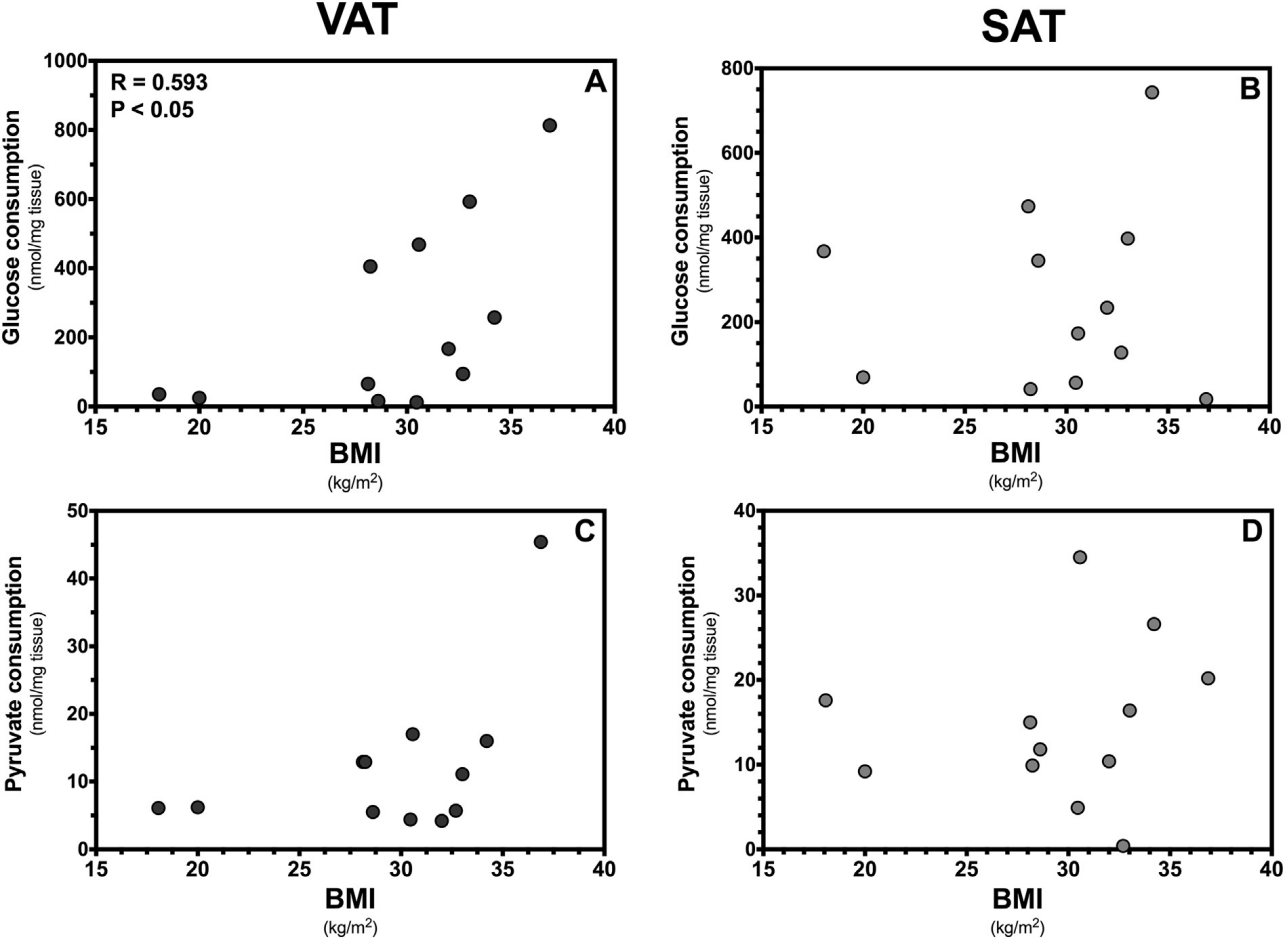
Substrate consumption by cultured visceral (VAT) and subcutaneous (SAT) adipose tissue. The figure shows glucose and pyruvate consumption by VAT (panel A, C) and SAT (panel B, D) of patients (n = 12, F:M, 9:3) with an average age of 46.8 years (range 26–83 years, mean) and different body mass index (BMI). The association between metabolite consumption and BMI was evaluated by computing Pearson correlation coefficients (r) assuming Gaussian distribution and a confidence interval of 95%. All *P* values < 0.05 were considered statistically significant.

Our data showed that glucose consumption by explants of VAT was positively correlated with the BMI of the patients (*r* = 0.593, *p* < 0.05) (Fig. 1A), illustrating that VAT explants collected from patients with higher BMI consumed more glucose than those collected from patients with lower BMI. Furthermore, this effect was observed in the absence of insulin and was thus independent of the glucose uptake mediated by the hormone. However, there was no correlation between pyruvate consumption by VAT explants and the patients BMI (Fig. 1C). Our data also showed that glucose and pyruvate consumption by SAT was independent of the patients BMI, illustrating that the metabolic functioning of this tissue is not affected by the BMI of the patients (Fig. 1B,D).

### 3.5 Secretome of SAT is not affected by the BMI of the patients but VAT production of alanine and lactate is negatively correlated with the patients BMI

**Fig. 2:**
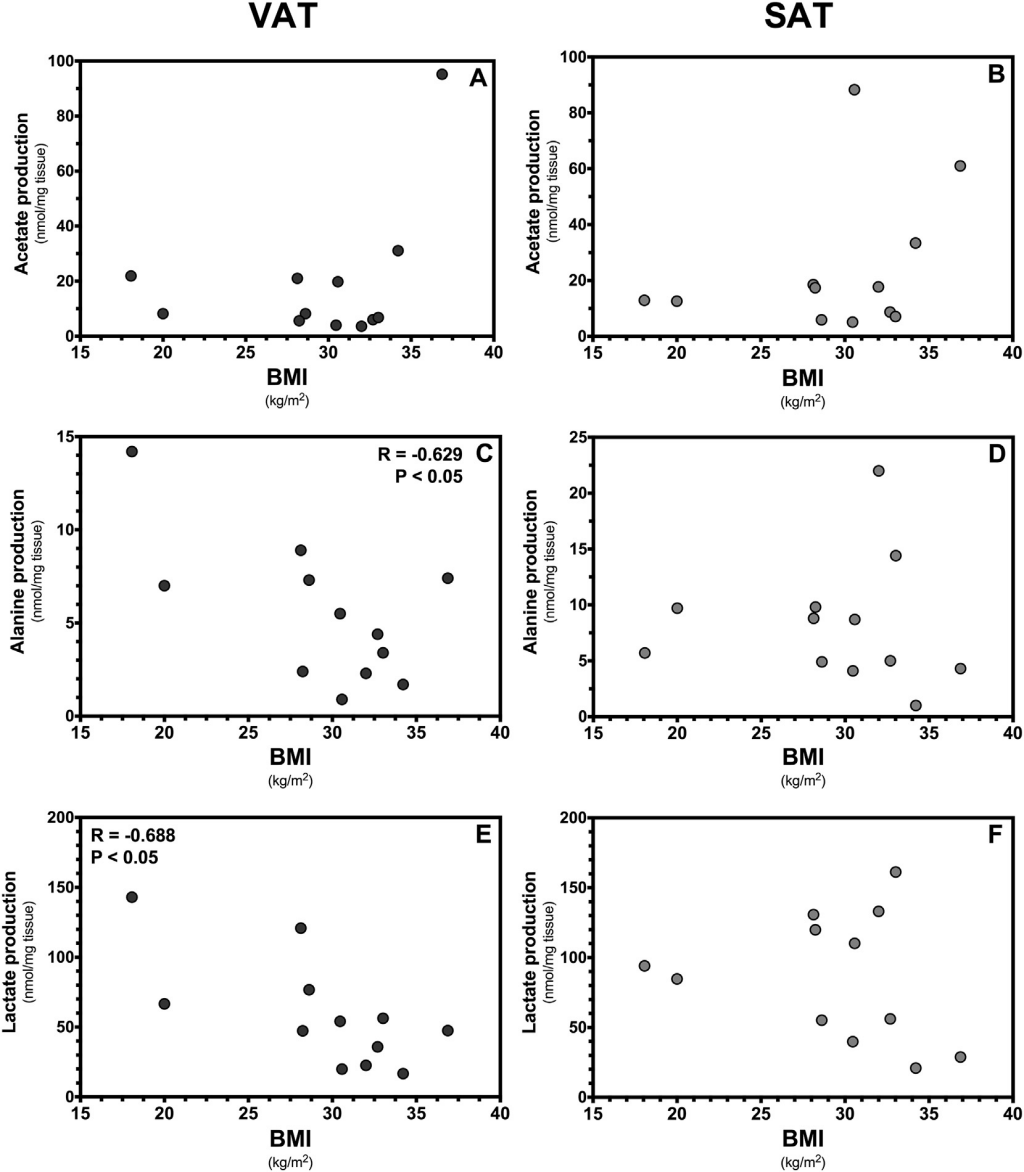
Substrate production by cultured visceral (VAT) and subcutaneous (SAT) adipose tissue. The figure shows acetate, alanine and lactate production by VAT (panels A, C, E) and SAT (panels B, D, F) of patients (n = 12, F:M, 9:3) with an average age of 46.8 years (range 26–83 years, mean) and different body mass index (BMI). The association between metabolite production and BMI was evaluated by computing Pearson correlation coefficients (r) assuming Gaussian distribution and a confidence interval of 95%. All *P* values < 0.05 were considered statistically significant.

Acetate production by both, VAT and SAT, was not correlated with the BMI of the patients (Fig. 2A, B). However, the production of alanine by VAT was negatively correlated (*r* = − 0.629, *p* < 0.05) with the patients BMI illustrating that alanine production was decreased in VAT of patients with higher BMI (Fig. 2C). Lactate production by VAT was also negatively correlated (*r* = − 0.688, *p* < 0.05) with the patients BMI (Fig. 2E). On the other hand, the production of acetate, alanine or lactate of SAT remains independent of the patients BMI (Fig. 2B, D, F). Overall, these results show that the metabolic behavior of VAT, but not SAT, in culture is dependent of the patients BMI.

## 4 Discussion

Adipose tissue undergoes dynamic remodeling in response to several nutritional and metabolic cues. Those alterations can be region-dependent and provide key information concerning distinguishing severity of obesity to health. Despite several differences have been described in the physiological properties of SAT and VAT, our results show that the two regional adipose tissue deposits appear to have a similar glucose metabolic profile when directly compared without considering the BMI of the patients. However, when we correlate the metabolic profile of VAT and SAT with the patient's BMI, our results show that BMI promotes a metabolic reprogramming of adipose tissue, particularly in the glycolytic profile of VAT, towards an increase in de novo lipogenesis. As this metabolic profiling was performed in the absence of insulin, which is known to have an important role in stimulating the cell glucose uptake, these preliminary results highlight that the metabolic reprogramming may occur beyond the effects known to be mediated by insulin. Visceral obesity is tightly connected with increased insulin resistance and secondary hyperinsulinism that are believed to have an important role in the metabolic shift experienced by obese patients. Thus, targeting insulin resistance and hyperinsulinism has been the mainstay to correct obesity related metabolic imbalance. What our data highlights is that beyond the effects that are known to be driven by insulin mediated pathways, there could be additional routes leading to the observed metabolic shifts, independent of hyperinsulinemia or insulin resistance.

**Fig. 3:**
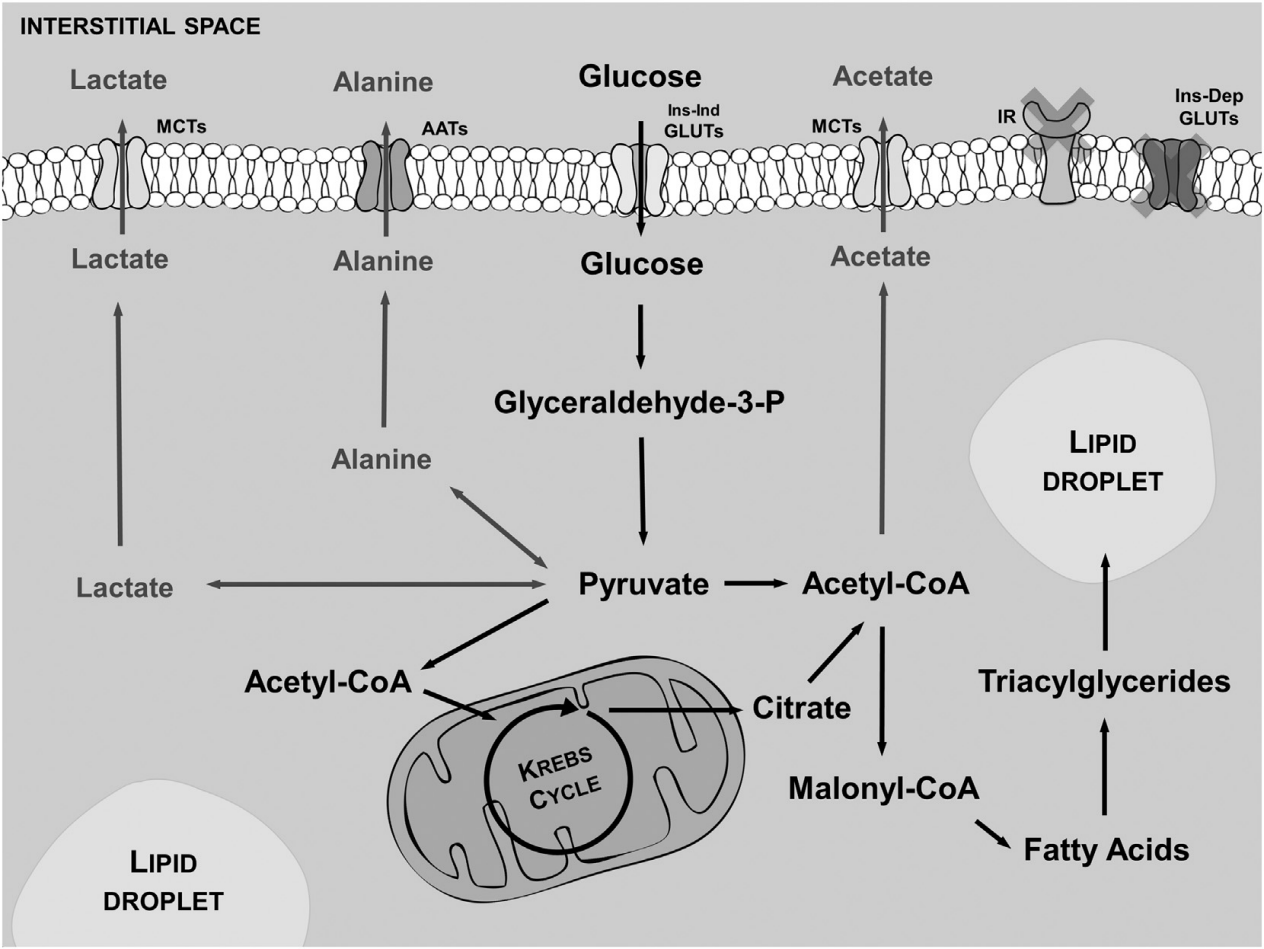
Schematic representation of the studied metabolic pathways in adipose tissue explants, visceral adipose tissue (VAT) and subcutaneous adipose tissue (SAT). We focused on glucose metabolism independent from insulin stimuli. Our results show that tissue explants of VAT from subjects with higher BMI consumed more glucose and produced less metabolites directly derived from pyruvate (e.g. lactate and alanine) and maintained the same production of acetate. Thus, we suggest that with the increase of BMI, VAT reprograms glucose metabolism towards de novo lipogenesis. Thus, our results provide a preliminary evidence that increased BMI promotes an insulin-independent metabolic reprogramming of glucose metabolism in VAT that may be associated with increased de novo lipogenesis. (AATs-Aminoacid transporters; Ins-Dep GLUTs-Insulin dependent glucose transporters; Ins-Ind GLUTs-Insulin independent glucose transporters; MCTs-Monocarboxylic acid transporters; IR-Insulin receptors).

Epidemiologic studies show that excess of VAT is more closely correlated with the development of metabolic disorders, such as type 2 diabetes and the metabolic syndrome, than excess of SAT [21]. One of the commonly accepted explanations for this observation is that the two fat depots are innately metabolically different [22]. This assumption was mostly derived from in vivo and ex vivo studies performed in the presence of insulin and using different metabolic approaches [21], [22]. When analyzing a pool of samples without considering the different BMI, our results show that this may not be the case since both, as VAT and SAT seem to have the same glucose metabolic profile. However, VAT and SAT may have different sensitivity to insulin [22], [23]. Several studies reported that insulin resistance is more associated with VAT mass than with obesity itself [24], [25] showing a possible regional variation in insulin sensitivity. Recently, it was shown that subcutaneous and omental adipocytes of non-obese subjects have no differences in what concerns insulin sensitivity [22], [26], raising the possibility that BMI may play a role on insulin sensitivity of these tissues. Using paired samples of VAT and SAT explants of patients with different BMI, we observed a metabolic reprogramming independent of the presence of insulin and thus, likely autonomous from insulin resistance or hyperinsulinemia. There was an increase of glucose consumption strongly associated with the increase of the patient's BMI. Notably, lactate and alanine production were negatively associated with the increase in the subjects BMI. Thus, glucose is redirected to other metabolic pathways than the production of these two metabolites. In humans, adipocytes are known to produce fatty acids and triglycerides from non-lipid precursors [27], [28], [29]. De novo lipogenesis is stimulated by overfeeding of carbohydrates [28], [30] and it was recently proposed that suppression of the machinery responsible for de novo lipogenesis constitutes a mechanism by which dietary fat competes with carbohydrates for storage as triglycerides in adipose tissue [31]. Our results provide clear evidence that in absence of insulin cultured VAT, but not SAT, increases the consumption of glucose, followed by a decrease in the production of lactate and alanine, illustrating that it may be redirected to de novo lipogenesis. Our results show that VAT is a metabolically active tissue that responds to BMI and extracellular glucose concentration. Tissue explants of VAT from subjects with higher BMI presented an insulin-independent metabolic reprogramming of glucose metabolism that may be associated with increased de novo lipogenesis, for all other metabolic routes evaluated were not found to be stimulated (summarized in Fig. 3), particularly lactate, alanine and acetate production. This suggests that several obesity-related metabolic shifts usually attributed to insulin action may be due to the metabolic adaptations of the tissue induced by the subjects BMI beyond insulin mediated effects. Further studies will be needed to test this hypothesis. The use of paired explants of VAT and SAT reflects a more physiological in vivo function since the tissue is relatively intact and there is no selective loss of certain types of adipocytes. Besides, it avoids the use of mechanical and/or biological digestions to isolate cells, which may alter the metabolic behavior of cells. However, the use of homogeneous preparations of isolated adipocytes may help to further understand the non-insulin mediated metabolic reprogramming of VAT during the increase of the subjects BMI.

In conclusion, our preliminary results show that BMI is associated with region-dependent metabolic reprogramming towards de novo lipogenesis in VAT and suggest that insulin action may not be required for the deleterious metabolic remodeling of VAT. Nevertheless, this data is still preliminary and studies are required to further test this hypothesis and to understand the role of insulin and the molecular mechanisms underlying this metabolic reprograming. Adipocyte is gaining attention as a central rheostat of systemic and localized energy homeostasis. Identification of the molecular mechanisms underlying VAT and SAT metabolic behavior, which promote lipid accumulation and VAT expansion in detriment of the more physiological and less detrimental lipid storage in the peripheral SAT, harbors the potential of disclosing novel molecular targets that could be used to counteract obesity related metabolic co-morbidities.

## Transparency document

Transparency document. Image 2

## References

[1] Laclaustra M., Corella D., Ordovas J.M. (2007). Metabolic syndrome pathophysiology: the role of adipose tissue. Nutr. Metab. Cardiovasc. Dis..

[2] Carey V.J., Walters E.E., Colditz G.A. (1997). Body fat distribution and risk of non-insulin-dependent diabetes mellitus in women the nurses' health study. Am. J. Epidemiol..

[3] Wang Y., Rimm E.B., Stampfer M.J., Willett W.C., Hu F.B. (2005). Comparison of abdominal adiposity and overall obesity in predicting risk of type 2 diabetes among men. Am. J. Clin. Nutr..

[4] Yusuf S., Hawken S., Ounpuu S. (2005). Obesity and the risk of myocardial infarction in 27 000 participants from 52 countries: a case-control study. Lancet.

[5] Hoffstedt J., Arner E., Wahrenberg H. (2010). Regional impact of adipose tissue morphology on the metabolic profile in morbid obesity. Diabetologia.

[6] Jankovic A., Korac A., Srdic-Galic B. (2014). Differences in the redox status of human visceral and subcutaneous adipose tissues–relationships to obesity and metabolic risk. Metabolism.

[7] Pischon T., Boeing H., Hoffmann K. (2008). General and abdominal adiposity and risk of death in Europe. N. Engl. J. Med..

[8] Manolopoulos K., Karpe F., Frayn K. (2010). Gluteofemoral body fat as a determinant of metabolic health. Int. J. Obes..

[9] Tran T.T., Yamamoto Y., Gesta S., Kahn C.R. (2008). Beneficial effects of subcutaneous fat transplantation on metabolism. Cell Metab..

[10] Abate N., Garg A., Peshock R., Stray-Gundersen J., Grundy S. (1995). Relationships of generalized and regional adiposity to insulin sensitivity in men. J. Clin. Invest..

[11] Hutley L., Prins J.B. (2005). Fat as an endocrine organ: relationship to the metabolic syndrome. Am. J. Med. Sci..

[12] Okamoto Y., Kihara S., Funahashi T., Matsuzawa Y., Libby P. (2006). Adiponectin: a key adipocytokine in metabolic syndrome. Clin. Sci..

[13] Thorne A., Lonnqvist F., Apelman J., Hellers G., Arner P. (2002). A pilot study of long-term effects of a novel obesity treatment: omentectomy in connection with adjustable gastric banding. Int. J. Obes. Relat. Metab. Disord..

[14] Kraunsøe R., Boushel R., Hansen C.N. (2010). Mitochondrial respiration in subcutaneous and visceral adipose tissue from patients with morbid obesity. J. Physiol..

[15] Liesenfeld D.B., Grapov D., Fahrmann J.F. (2015). Metabolomics and transcriptomics identify pathway differences between visceral and subcutaneous adipose tissue in colorectal cancer patients: the ColoCare study. Am. J. Clin. Nutr..

[16] Fain J.N., Madan A.K., Hiler M.L., Cheema P., Bahouth S.W. (2004). Comparison of the release of adipokines by adipose tissue, adipose tissue matrix, and adipocytes from visceral and subcutaneous abdominal adipose tissues of obese humans. Endocrinology.

[17] Ibrahim M.M. (2010). Subcutaneous and visceral adipose tissue: structural and functional differences. Obes. Rev..

[18] Insenser M., Montes-Nieto R., Vilarrasa N. (2012). A nontargeted proteomic approach to the study of visceral and subcutaneous adipose tissue in human obesity. Mol. Cell. Endocrinol..

[19] Martins A.D., Moreira A.C., Sa R. (1852). Leptin modulates human Sertoli cells acetate production and glycolytic profile: a novel mechanism of obesity-induced male infertility?. Biochim. Biophys. Acta.

[20] Hu J., Kyrou I., Tan B.K. (2016). Short-chain fatty acid acetate stimulates adipogenesis and mitochondrial biogenesis via GPR43 in brown adipocytes. Endocrinology.

[21] Kissebah A.H., Krakower G.R. (1994). Regional adiposity and morbidity. Physiol. Rev..

[22] Westergren H., Danielsson A., Nystrom F.H., Stralfors P. (2005). Glucose transport is equally sensitive to insulin stimulation, but basal and insulin-stimulated transport is higher, in human omental compared with subcutaneous adipocytes. Metabolism.

[23] Zierath J.R., Livingston J.N., Thorne A. (1998). Regional difference in insulin inhibition of non-esterified fatty acid release from human adipocytes: relation to insulin receptor phosphorylation and intracellular signalling through the insulin receptor substrate-1 pathway. Diabetologia.

[24] Fujioka S., Matsuzawa Y., Tokunaga K., Tarui S. (1987). Contribution of intra-abdominal fat accumulation to the impairment of glucose and lipid metabolism in human obesity. Metabolism.

[25] Despres J.P., Nadeau A., Tremblay A. (1989). Role of deep abdominal fat in the association between regional adipose tissue distribution and glucose tolerance in obese women. Diabetes.

[26] Stolic M., Russell A., Hutley L. (2002). Glucose uptake and insulin action in human adipose tissue–influence of BMI, anatomical depot and body fat distribution. Int. J. Obes. Relat. Metab. Disord..

[27] Roberts R., Hodson L., Dennis A.L. (2009). Markers of de novo lipogenesis in adipose tissue: associations with small adipocytes and insulin sensitivity in humans. Diabetologia.

[28] Aarsland A., Chinkes D., Wolfe R.R. (1997). Hepatic and whole-body fat synthesis in humans during carbohydrate overfeeding. Am. J. Clin. Nutr..

[29] Letexier D., Pinteur C., Large V., Frering V., Beylot M. (2003). Comparison of the expression and activity of the lipogenic pathway in human and rat adipose tissue. J. Lipid Res..

[30] Minehira K., Vega N., Vidal H., Acheson K., Tappy L. (2004). Effect of carbohydrate overfeeding on whole body macronutrient metabolism and expression of lipogenic enzymes in adipose tissue of lean and overweight humans. Int. J. Obes. Relat. Metab. Disord..

[31] Marcelino H., Veyrat-Durebex C., Summermatter S. (2013). A role for adipose tissue de novo lipogenesis in glucose homeostasis during catch-up growth: a Randle cycle favoring fat storage. Diabetes.

